# Subscapular Fossa Osteochondroma: A Case Report of an Anatomical Surgical Approach

**DOI:** 10.1155/cro/5541056

**Published:** 2026-08-17

**Authors:** Carina Codau, Albert Ferrando

**Affiliations:** ^1^ Department of Orthopedic Surgery, Hospital Universitari Sant Joan de Reus, Tarragona, Spain, hospitalsantjoan.cat

**Keywords:** case report, medial paravertebral approach, osteochondroma, scapular osteochondroma, subscapular fossa

## Abstract

**Introduction:**

Subscapular osteochondromas are rare lesions, and reports describing their management through the medial paravertebral longitudinal approach are scarce. This case highlights the surgical management of a giant symptomatic osteochondroma with suspected malignant transformation using a muscle‐sparing approach that provided excellent functional recovery. Osteochondromas are the most common benign bone tumors and rarely involve the scapula. Although most remain asymptomatic, progressive pain, functional limitation, and cartilage cap thickening may raise suspicion of malignant transformation and justify surgical excision.

**Case Presentation:**

A 60‐year‐old woman was diagnosed with an incidental osteochondroma arising from the medial aspect of the subscapular fossa. After 10 years of conservative management, she developed progressive pain and functional limitation. Imaging demonstrated a 54 × 40 × 59 mm lesion with a cartilage cap thicker than 22 mm, raising suspicion of malignant transformation. The tumor was excised through Elhassan′s medial paravertebral longitudinal approach. Histopathological examination confirmed a benign osteochondroma. At 1‐year follow‐up, the patient achieved excellent functional outcomes with a Constant score of 96, an ASES score of 98.3, an SSV of 100%, complete symptom resolution, and no evidence of recurrence.

**Conclusion:**

This case demonstrates that the medial paravertebral longitudinal approach provides safe exposure for giant osteochondromas of the subscapular fossa while minimizing soft‐tissue disruption. Progressive symptoms and cartilage cap thickening should prompt evaluation for possible malignant transformation, even after years of apparently benign evolution.

## 1. Introduction

Osteochondromas are the most common bone pseudotumor (15% of all bone tumors, 45.3% of benign tumors, and 3% in the general population) [1]. Solitary lesions are typically sporadic, whereas multiple lesions suggest hereditary multiple exostoses (HMEs), an autosomal dominant condition associated with EXT1 and EXT2 gene mutations [2, 3]. These tumors develop in bones formed by endochondral ossification, most commonly affecting the distal femur, proximal tibia, and humerus (90%). The scapula and pelvis are atypical locations (3%–4.6%) [4].

They usually present during the first three decades of life and are reported more frequently in males, although some authors report no gender differences [5]. Therefore, symptomatic lesions arising in a 60‐year‐old woman are distinctly uncommon and should prompt careful evaluation, particularly when progressive symptoms or imaging findings raise concern for malignant transformation.

Clinical manifestations range from asymptomatic to pain, restricted movement, vascular compression, snapping scapula syndrome or scapular winging, and exophytic masses [6]. At the acromioclavicular level, they can cause subacromial impingement of the rotator cuff [4]. Rarely, scapular osteochondromas may produce neurovascular compression or brachial plexopathy [2].

Diagnosis is usually clinical and radiographic. In flat bones like the scapula, ultrasound, CT, or MRI may be required. Treatment is usually conservative. Surgery is indicated in symptomatic cases, postskeletal maturity growth, vascular proximity, fractures, or suspicion of malignancy. Prognosis after excision is favorable, and recurrence is rare (< 5%).

Although several surgical approaches have been described for scapular osteochondromas, there is very limited literature regarding the use of Elhassan′s medial paravertebral longitudinal approach for tumor excision. Originally developed for lower trapezius tendon transfer, this muscle‐sparing approach may provide excellent exposure of the subscapular fossa while minimizing soft‐tissue disruption. We present a case of a giant symptomatic osteochondroma with radiological suspicion of malignant transformation successfully treated using this technique, together with its surgical description and clinical outcomes.

## 2. Case Presentation

A 60‐year‐old woman with a medical history of subclinical hypothyroidism was referred in 2013 after an incidental radiographic finding of a calcified lesion in the right thoracoscapular region. She worked in a clothing store, had a normal body habitus (although exact height, weight, and body mass index [BMI] measurements were not available in the medical records), was a nonsmoker, and reported no alcohol consumption. There was no known family history of HMEs, osteochondromas, or other bone tumors. Thoracic CT revealed a pedunculated bony lesion arising from the medial aspect of the right subscapular fossa, compatible with an osteochondroma, and MRI confirmed the diagnosis without radiological signs of malignant transformation (Figure 1).

Figure 1(A) Computed tomography in the coronal, sagittal, and axial planes demonstrated a pedunculated exophytic lesion arising from the medial aspect of the subscapular fossa, compatible with a giant osteochondroma measuring 54 × 40 × 59 mm, with an 11.8‐mm pedicle base and a cartilage cap measuring more than 22 mm in thickness. The hallmark feature was uninterrupted cortical and medullary continuity between the lesion and the native scapula, consistent with an osteochondroma. Three‐dimensional reconstruction further illustrated the relationship between the tumor and the thoracic cage and facilitated preoperative planning. (B) Axial T2‐weighted magnetic resonance imaging demonstrates a subscapular osteochondroma arising from the subscapular fossa, measuring 54 × 40 mm, with a pedunculated base of 11.8 mm. The second image shows a cartilage cap measuring 22 mm in thickness.

**Figure:**
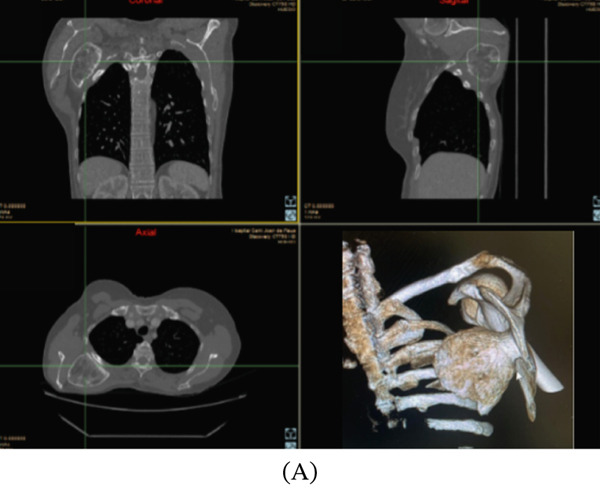
(A)

**Figure:**
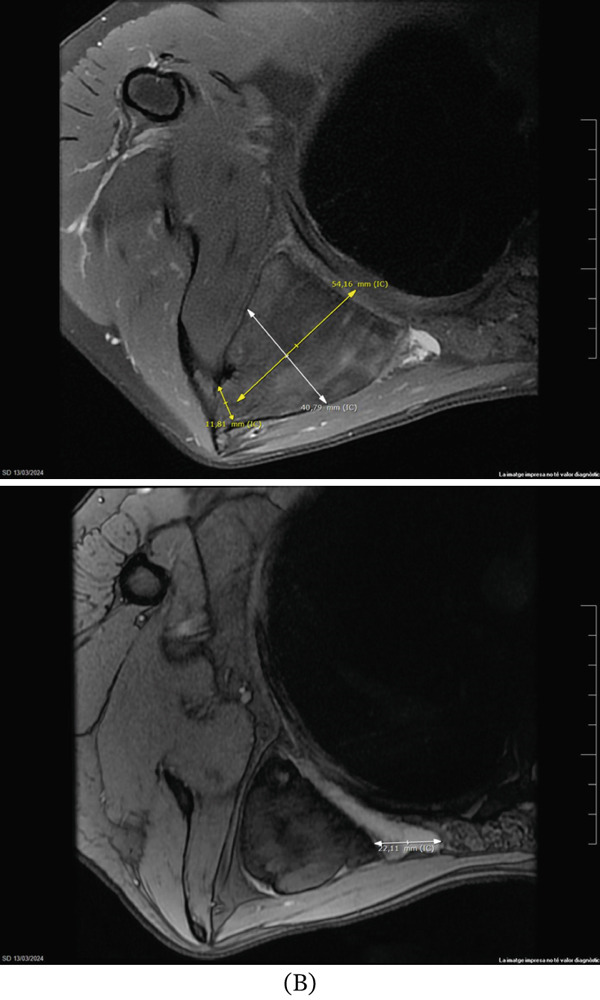
(B)

Clinically, at the initial assessment in 2013, the patient had full, painless shoulder range of motion without functional limitation; the only symptom was an occasional snapping sensation during scapulothoracic movement. No palpable mass was detected on physical examination because of the deep location of the lesion within the subscapular fossa. Exact range‐of‐motion measurements were not recorded in the medical records. Because her symptoms had minimal impact on her daily activities and work, she preferred conservative management and declined surgery.

Follow‐up consisted of clinical assessment every 6 months and annual imaging. During the COVID‐19 pandemic, follow‐up was interrupted at the patient′s request. She returned in 2023 because of progressive pain, worsening shoulder function, and increasing concern regarding her symptoms. The active shoulder range of motion showed forward flexion of 170°, external rotation of 40° (symmetric to the contralateral side), and internal rotation to the T12 vertebral level. The scapular snapping persisted, but no clinical scapular winging or obvious scapular dyskinesis was observed. Neurovascular examination of the affected upper limb was normal, with no motor or sensory deficits and no evidence of vascular compromise. Preoperative functional assessment showed an ASES score of 76 points and an SSV of 80%.

Repeat MRI demonstrated a giant osteochondroma measuring 54 × 40 × 59 mm, with an 11.8‐mm pedicle base and a cartilage cap measuring more than 22 mm in thickness, raising suspicion of malignant transformation. After discussing treatment options, surgery was chosen.

**Figure 2:**
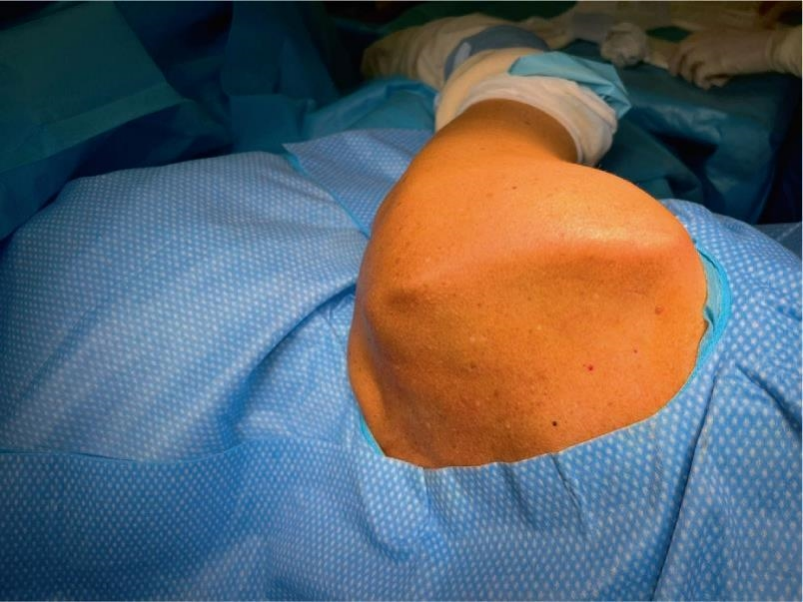
Patient position in the left lateral decubitus for surgical intervention.

**Figure 3:**
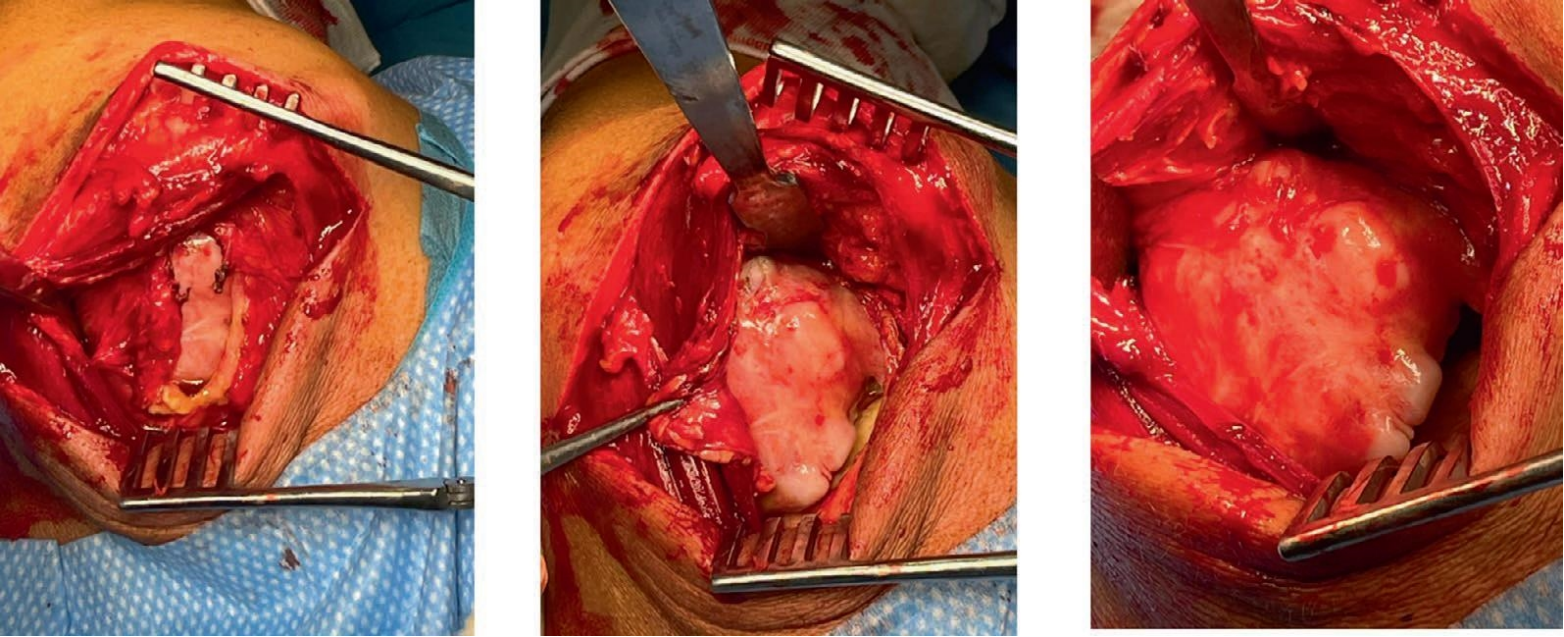
Surgical approach through the triangular space between the lower trapezius, latissimus dorsi, and medial border of the scapula with the osteochondroma in the subscapular fossa.

In 2024, the patient underwent surgery. General anesthesia was combined with an interscalene block for postoperative shoulder analgesia and an erector spinae plane block to improve analgesia of the posterior thoracic wall, given the medial scapular incision and scapulothoracic dissection required for tumor excision. She was placed in the left lateral decubitus position (Figure 2). The medial paravertebral longitudinal approach was selected because it provides direct access to lesions arising from the medial subscapular fossa through a muscle‐sparing internervous plane. This avoided detachment of the trapezius and extensive elevation of the posterior scapular musculature required by more extensile posterior approaches while allowing safe en bloc excision of the tumor. The intermuscular and internervous plane between the lower trapezius and latissimus dorsi was identified (Figure 3). The lower trapezius was retracted, and with the arm in full adduction and internal rotation, the rhomboid major was retracted proximally without detachment. A longitudinal incision was made in the synovial capsule overlying the osteochondroma. The tumor pedicle was identified and resected with saws and chisels. The surgical specimen was sent for pathology. The site was thoroughly irrigated with saline, and two deep drains were placed to prevent postoperative hematoma. The wound was closed with subcutaneous sutures and skin staples, followed by a compressive dressing.

Postoperative radiographs and clinical course were unremarkable. Drains were removed at 72 h, and the patient was discharged with a sling for 2 weeks.

The wound healed uneventfully. Staples were removed at 2 weeks, and the patient began a rehabilitation program with active scapular movement and strengthening exercises (resisted flexion, isometric bands, and trapezius strengthening) and postural correction due to kyphotic posture and right scapular elevation.

**Figure 4:**
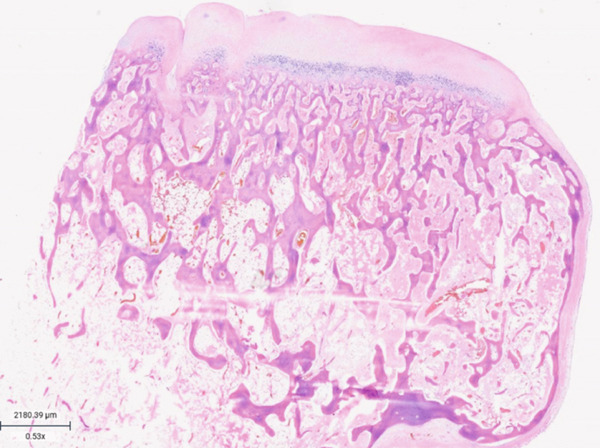
Histopathological examination of the surgical specimen showing chondrocytes arranged linearly without nuclear atypia and absence of necrosis and binucleation of cells.

The histological report described a single tissue fragment measuring 80 × 60 × 50 mm, osseous and polypoid, with a multinodular, smooth, whitish surface of cartilaginous appearance. The base measured 30 mm with an osseous appearance. On sectioning, the cartilaginous cap measured approximately 25 mm thick without macroscopic alterations. Microscopic analysis revealed chondrocytes arranged linearly without nuclear atypia and absence of necrosis and binucleation of cells (Figure 4).

**Figure 5:**
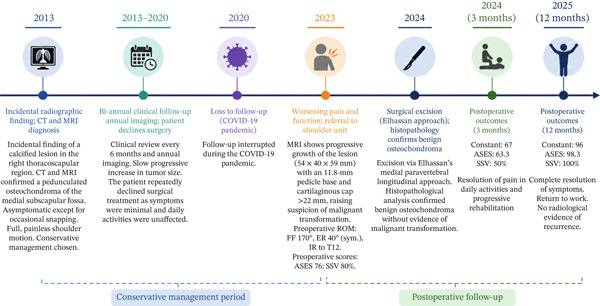
Timeline of the patient′s clinical course.

Postoperative radiographs confirmed complete excision of the lesion without evidence of fracture, residual osseous lesion, or other complications. At 3 months, the patient had resumed activities of daily living without pain at rest and reported only mild discomfort during overhead activities (maximum pain of 6/15 with shoulder elevation). Functional scores were Constant 67, ASES 63.3, and SSV 50%. At the latest follow‐up (12 months), the patient had returned to her previous occupation without restrictions, reported complete resolution of symptoms, achieved a Constant score of 96, an ASES score of 98.3, and an SSV of 100%. Serial radiographs demonstrated no evidence of local recurrence or postoperative complications (Figure 5).

## 3. Discussion

Although osteochondromas are uncommon and often asymptomatic, early diagnosis helps prevent complications. Radiographically, pathognomonic continuity of the cortical and medullary bone confirms the diagnosis, as in this case. However, MRI is the best imaging modality for assessing surrounding structures, associated complications, and the nonmineralized cartilage cap, which shows low signal on T1 and high signal on T2 due to water content, allowing accurate measurement. A hyaline cartilage cap < 1 cm is considered normal, whereas > 2 cm in adults or > 3 cm in children suggests possible low‐grade chondrosarcoma and requires monitoring [2, 7]. Nevertheless, cartilage cap thickness alone is insufficient to establish malignant transformation, as demonstrated in the present case. Histopathological examination remains the diagnostic gold standard. In the case presented, although the cartilage cap exceeded the commonly accepted MRI threshold (thicker than 20 mm) for suspicion of secondary peripheral chondrosarcoma, histopathological examination demonstrated preserved enchondral ossification, orderly chondrocyte arrangement, absence of nuclear atypia, binucleation, necrosis, or permeative growth, confirming a benign osteochondroma. This case illustrates that cartilage cap thickness alone should not be considered diagnostic of malignant transformation and must always be interpreted in conjunction with clinical, radiological, and histopathological findings.

Peripheral chondrosarcoma (secondary to osteochondroma, primary synovial chondromatosis, or solitary/multiple enchondromas as in Ollier disease or Maffucci syndrome) [8] is rare but the most serious complication. It is the third most common primary malignant bone tumor (20%–27%) after multiple myeloma and osteosarcoma [2, 7]. It typically affects patients over 40 and has a worse prognosis when located in the pelvis or shoulder. Surgical margins must be wide due to resistance to chemotherapy and radiotherapy.

In this case, although malignant transformation was suspected because of progressive symptoms and cartilage cap thickening, CT and MRI provided sufficient anatomical and biological information for surgical planning. Since the lesion was considered resectable irrespective of the final diagnosis, additional investigations such as PET‐CT or image‐guided biopsy were not considered necessary, and definitive diagnosis was established by complete surgical excision and histopathological examination.

The differential diagnosis included secondary peripheral chondrosarcoma, parosteal osteosarcoma, and synovial osteochondromatosis. Corticomedullary continuity with the parent bone strongly supported the diagnosis of osteochondroma. The absence of aggressive periosteal reaction, mineralization pattern typical of parosteal osteosarcoma, multiple intra‐articular cartilaginous bodies, or infiltrative soft‐tissue extension made the alternative diagnoses unlikely. Definitive diagnosis was established by histopathological examination.

Histologic diagnosis of chondrosarcoma is difficult because of the borderline distinction between benign cartilage cells and low‐grade malignancy. Both macroscopic and microscopic analysis are mandatory. In cases of malignant transformation, the cartilage cap macroscopically can be thick and irregular. On microscopic analysis, according to Wuisman et al., histopathological diagnosis of chondrosarcoma should include increased cellularity, enlarged chondrocyte nuclei, double‐nucleated cells, and infiltrative, permeative growth of the lesion (medullary invasion, thinning, and invasion of the cortical wall) [9]. In our case, there were no macroscopic or microscopic criteria of chondrosarcoma, and a segmental resection was carried out successfully.

Management of osteochondromas is usually observational. Surgery is indicated in symptomatic cases or when malignancy is suspected; pedunculated lesions are excised directly, whereas sessile lesions require curettage to remove the cartilage cap completely and prevent recurrence. In our case, approximately 10 years passed before symptoms led to a change in treatment. Surgical resection—either open or arthroscopic—has been shown to be effective, with low recurrence rates and excellent functional recovery. In our case, due to the atypical location and size, an open approach was preferred; there are many surgical techniques:

A. Open resection: for large or deep lesions. Offers complete visualization and low recurrence but slower recovery.
B. Arthroscopy: for small, superficial lesions. Less pain, quicker recovery, and high functionality. Only appropriate when benignity is confirmed, as arthroscopic fluid could potentially disseminate malignant cells [2, 3, 6].
C. Trans‐scapular approaches: include Judet′s incision, modified Judet, and Brodsky′s lateral column approach, among others. Used in complex scapular fractures. Breglia et al. reported excellent functional outcomes with return to full activity at 6 months. However, these are aggressive techniques with a high risk of injury to the suprascapular nerve and circumflex scapular artery [10].

In this case, the medial paravertebral longitudinal approach described by Elhassan et al. (originally used for lower trapezius transfer in irreparable rotator cuff tears [11]) was selected because the tumor arose from the medial aspect of the subscapular fossa, where this approach provides direct access through an internervous and intermuscular plane while preserving the trapezius, rhomboids, and posterior deltoid. The skin incision and medial paravertebral interval described by Elhassan et al. for lower trapezius tendon transfer were preserved; the intermuscular plane between the lower trapezius and latissimus dorsi was developed as originally described, and the rhomboid major was retracted without detachment. Unlike the original technique, however, no tendon harvest or transfer was performed. Instead, after exposing the ventral surface of the scapula, the overlying bursa was opened longitudinally, the osteochondroma pedicle was identified, and the tumor was excised en bloc using an oscillating saw and osteotomes.

Previous reports have described successful open excision of subscapular osteochondromas using extensile posterior approaches (one must split the trapezius and deltoid fibers and then all the infraspinatus muscle must be lifted off the infraspinatus fossa to allow lifting the scapula from the chest wall [1]). Alatassi et al. [1] reported complete tumor excision with resolution of scapular winging through a conventional posterior approach, whereas Breglia et al. [10] described favorable functional outcomes following open resection of a symptomatic subscapular osteochondroma. However, both reports relied on more extensive posterior exposures. In contrast, the present case demonstrates that Elhassan et al.′s medial paravertebral longitudinal approach provides adequate exposure of the ventral scapular surface through a muscle‐sparing intermuscular interval, allowing complete tumor excision while minimizing soft‐tissue disruption and facilitating early rehabilitation.

Despite the patient′s age, prolonged clinical evolution, atypical scapular location, and cartilage cap exceeding 20 mm, imaging did not demonstrate cortical destruction, soft‐tissue invasion, or other aggressive radiological features suggestive of high‐grade malignancy. Moreover, the lesion appeared technically resectable en bloc with preservation of the surrounding scapular structures. Therefore, marginal excision was considered appropriate to obtain a definitive diagnosis while minimizing surgical morbidity. Histopathological examination subsequently confirmed the absence of malignant transformation, validating this treatment strategy.

Thus, by using this less invasive medial paravertebral longitudinal approach that we have described, one can begin the rehabilitation program sooner and achieve better functional results: pendular motion by day 2, rotation and abduction at 3 weeks, and full pain‐free mobility at 12 months. There are no standardized postoperative protocols after scapular tumor excision. The goal is to strengthen the periscapular musculature and restore joint balance. In this case, the rehabilitation program began at 2 weeks after surgery. The greatest improvement in function is seen after the 3‐month mark, when the patient has regained full motion and has begun strengthening exercises. It is at this point that symmetric scapular exercises are key to regaining shoulder confidence and function.

Postoperative MRI was not routinely performed because complete surgical excision was achieved and histopathological examination confirmed a benign osteochondroma without features of malignant transformation. Furthermore, the patient became completely asymptomatic, achieved excellent functional recovery, and serial clinical and radiographic follow‐up showed no evidence of recurrence. Therefore, additional MRI surveillance was not considered clinically necessary.

Genetic testing was not performed because the patient presented with a solitary osteochondroma, had no known family history of HMEs, and showed no clinical or radiological features suggestive of this syndrome. Therefore, routine EXT1/EXT2 testing was not considered indicated.

Although the patient remained asymptomatic without radiographic evidence of recurrence at 1 year, longer term surveillance would be desirable, particularly considering the initial radiological suspicion of malignant transformation. This represents a limitation of the present report.

## 4. Conclusions

Scapular osteochondromas are rare benign tumors, but progressive symptoms, enlargement of the lesion, and cartilage cap thickening should raise suspicion of malignant transformation and prompt surgical treatment. This case illustrates that cartilage cap thickness alone should not be considered diagnostic of chondrosarcoma, as definitive diagnosis ultimately relies on histopathological examination. Complete excision of the cartilaginous cap remains essential to minimize the risk of recurrence. The medial paravertebral longitudinal approach described by Elhassan et al. provided safe and direct access to the medial subscapular fossa through a muscle‐sparing interval, allowing complete tumor excision with excellent functional recovery. Although no gold standard surgical approach currently exists, this technique represents a valuable alternative for appropriately selected patients. Longer term follow‐up and additional studies are needed to validate its reproducibility and define its role in the surgical management of ventral scapular tumors.

**Nomenclature**
ASES: American Shoulder and Elbow Surgeons
CT: computed tomography
HMEs: hereditary multiple exostoses
MRI: magnetic resonance imaging
SSV: subjective shoulder value

## Author Contributions

A.F. diagnosed the patient and decided on surgical treatment when indicated. A.F. performed the surgical procedure. C.C. conducted the research on scapular osteochondroma and wrote the article for publication. A.F. and C.C. reviewed and approved the text for publication.

## Funding

No funding was received for this manuscript.

## Consent

Written informed consent was obtained from the patient for the publication of this case report and accompanying images.

## Conflicts of Interest

The authors declare no conflicts of interest.

## General Statement

It is imperative to perform a thorough clinical and radiological evaluation of these tumors to identify potentially malignant lesions. In addition, it is of equal importance to confirm the definitive diagnosis through histopathological examination.

## Data Availability

The data that support the findings of this study are available from the corresponding author upon reasonable request.
